# Thickness of the human cranial diploe in relation to age, sex and general body build

**DOI:** 10.1186/1746-160X-1-13

**Published:** 2005-12-20

**Authors:** Niels Lynnerup, Jacob G Astrup, Birgitte Sejrsen

**Affiliations:** 1 Laboratory of Biological Anthropology, Institute of Forensic Medicine, University of Copenhagen, Denmark

## Abstract

**Background:**

Earlier studies have addressed the human total cranial vault thickness and generally found no correlation with sex, age or body weight. However, the thickness of the diploe has not been investigated. Our study has determined the diploeic thickness of the human cranial vault using modern autopsy material.

**Methods:**

The diploeic bone thickness was measured in 64 individuals (43 males, 21 females) autopsied at our institute. The thickness was measured by X-raying biopsies trephined at four specific locations on the skull. Complete medical records and pathologic autopsy results were available.

**Results:**

There was a statistically significant difference in diploeic thickness between males and females in the frontal region only. Diploeic thickness was highly correlated with total cranial vault bone thickness, except for the left euryon in females. Subsequent analyses failed to reveal any correlations between the diploeic thickness and age and height and weight of the individual.

**Conclusion:**

Males overall have a thicker diploe, albeit this difference is statistically significant only in the frontal region. We could not discern any trends as pertains to diploeic thickness versus age, height or weight. Since the thickness of the diploe may be an important parameter in biomechanical modelling of the cranial vault, this means that the diploe can be built into such models based on the total cranial thickness, except for the frontal region where the sexual dimorphism must be taken into account. Our findings are consistent with previous studies relating the total cranial thickness to the same parameters, in that we found a high correlation between diploeic and total cranial thickness (except at the left euryon for females). Finally, we recommend that future studies try to incorporate CT or MR scan imaging, rather than point sampling, in order to achieve a total assessment of the dimensionalities of the diploe.

## Background

**Figure 1:**
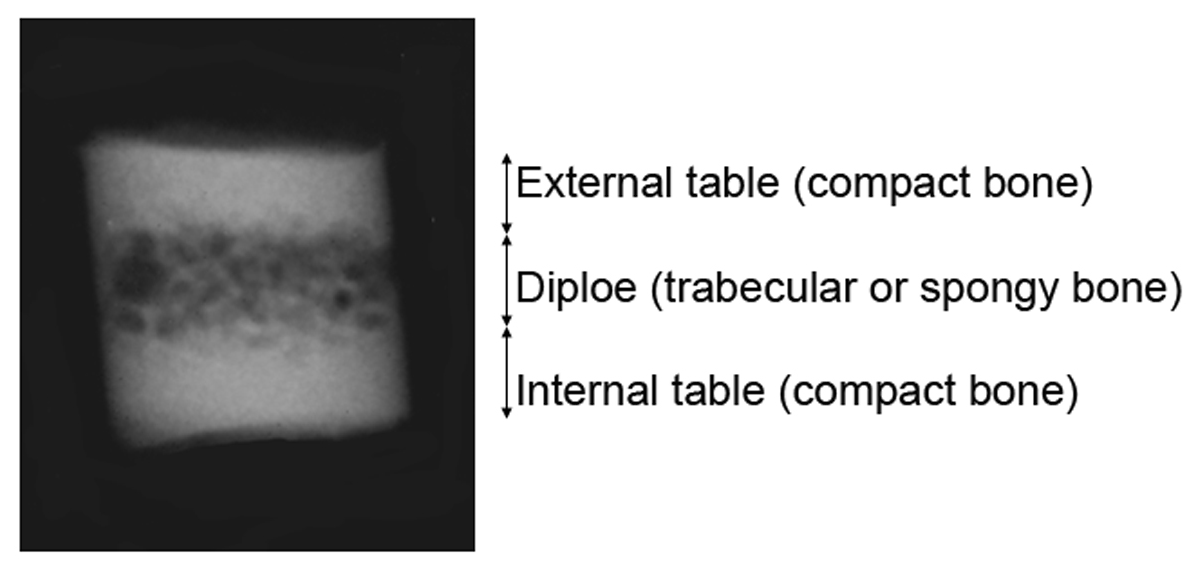
**X-ray of a biopsy**. The three bone layers of the cranial vault are indicated.

While the thickness of the human cranial vault has been investigated before, not least in terms of the relationship between cranial thickness and sex, age and general body build [1-11], these studies have mostly addressed the total thickness (diploe and the external and internal table). The main incentive for those studies has been to determine whether cranial vault thickness could be used as an indicator of sex and age. However, no clear trends have emerged, and the results have been somewhat conflicting. We wanted to analyse whether trends useful for sexing and ageing might emerge if we focused on the diploe (the cancellous or spongy bone within the laminae, or tables, of the vault bones of the skull [12] (see figure 1)). A few other studies have addressed the diploeic thickness, but these studies have mainly dealt with issues of cranial reconstructive surgery [13,14].

Diploeic and cranial thickness is an important variable to consider when carrying out biomechanical modelling of the skull. This has become an interesting venue of research, e.g. in terms of modelling cranial fractures in forensic pathology [15], and more detailed material properties of the human skull lately been presented, including the cross-sectional proportions of compact and cancellous bone [16,17].

We used X-rays to visualise the bone components. We used the same samples as in a previous study: a modern forensic material with complete autopsy results and antemortem medical information [11]. This also allowed us to compare the measures taken physically of the bone samples in the previous study with the measures taken from analyses of the X-rays.

While the external cranial surface is overall fairly smooth (at least above the linea nuchae and temporal-masseter line), the internal surface is much more irregular. This will of course have implications for measurements taken directly with calipers on cut margins, or on trephined specimens or when measuring thickness on cranial X-rays. Cranial thickness has been measured by X-rays in numerous studies, but often employing indirect projections or just using lateral projections [3,18-22]. There is probably no way to rigorously standardize cranial thickness measurements. However, we feel that our method of X-raying trephined bone biopsies is superior to analyses of, e.g., lateral cranial X-rays, as the bone structures are visualised in a perpendicular view without juxtapositional structures (see figure 1).

## Methods

The biopsies were obtained from 64 autopsied individuals at the Institute of Forensic Medicine, University of Copenhagen. The material consisted of cranial vault bone biopsies from 43 males (age range: 16 – 90, mean ± 1 S.D. = 48 ± 17 years) and 21 females (age range: 23 – 84 years, mean ± 1 S.D. = 48 ± 16 years). The biopsies were taken sequentially over a 6 month period, although selection by age was made to ensure a reasonable spread in age over adult ages. Cases with cranial trauma were excluded due to the forensic pathological exigencies of these cases.

The cranial vault biopsies were taken from four sites on each individual: (1) 1 cm in front of the bregma; (2) 1 cm behind lambda; (3) left euryon and (4) right euryon. Determination of the euryon sampling points was made visually. The biopsies were made with a 5 mm trephine perpendicular to the outer plane. The specimens were stored in coded, separate containers in 97% alcohol. In our previous study [11] the thickness of each specimen had been measured without knowledge of sex or age using a digital caliper connected to a computer [23]. In this study the specimens were X-rayed using a Siemens Dentotime ^® ^equipped with a Heliodent 70 tubus (exposure settings: 70 kV and 7mA). A coin, precisely 10 mm in diameter, was placed alongside the single biopsy for calibration purposes. The X-rays were analysed using the software associated with the digital X-ray equipment (VixWin32 by Gendex Imaging^®^). The software allowed for image calibration and morphometric analyses, whereby we measured the thickness of the diploe; the compact bone of the inner and outer plate; and total cranial thickness (figure 1). All measures were made without knowledge of sex or age. As a control, we compared our previous results (total cranial thickness measured with a calliper) with the present data (total cranial thickness measured on digital X-ray).

Autopsy finds and ante-mortem medical data were available, indicating that 27 cases had a history of, and autopsy finds consistent with, chronic drug and alcohol abuse. There were no cases with recognized bone or craniofacial diseases. Height and weight of the individuals was also recorded.

Mann-Whitney test was used to analyze for between-group differences and Pearson correlation tests were used to analyze correlations between diploeic thickness measures, age, height and weight. Scatterplots with LOWESS smoothing were used for graphical analyses of trends in cranial diploeic thickness vs. sex [10,24].

## Results

There were no statistically significant differences between the age makeup of the male and female subsamples (p = 0.937). There were no statistically significant differences in the thickness measures (including both total thickness and diploeic thickness) between the 27 cases with a history of chronic drug and alcohol abuse (19 males and 8 females) and the cases without such a history (24 males and 13 females). Consequently, all cases were used in the subsequent analyses.

Delineation between the compact bone and cancellous bone was not possible in 19 single biopsies (9 occipital; 2 frontal; 4 left and 4 right euryon, out of a total of 256 biopsies). When we compared the total cranial thickness as determined in our previous study [11] with the present and we found (expected) high correlations for all four sites (Pearson correlation coefficients: Frontal: r = 0.972; Occipital: r = 0.971; Right euryon: r = 0.920; and Left euryon: r = 0.962).

**Figure 2:**
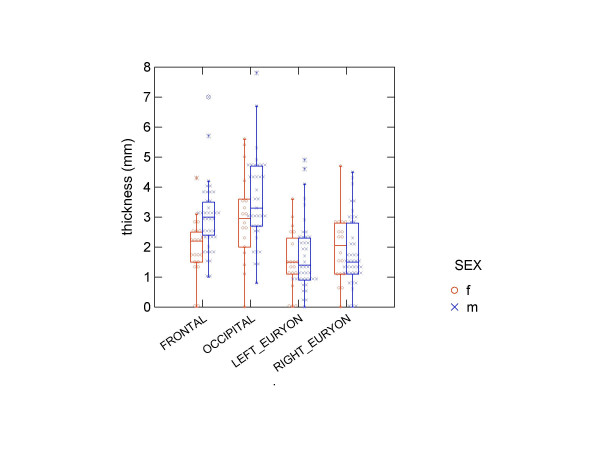
**Plot showing values for diploeic thickness at the four sampling points**. F:frontal; O: occipital; R: right euryon; and L: left euryon (o = females, x = males).

We found no statistically significant differences between males and females for the diploeic thickness, except for the frontal biopsies where there was a statistically significant difference with males having the thickest measures (table 1 and figure 2). It may be noted that males overall had the thickest diploeic bone layer also occipitally and at the left euryon, albeit not statistically significant.

**Table 1:** Summary statistics and significance tests (Mann-Whitney) for diploeic thickness measures by sex.

Sampling point	n	Mean (mm)	Std. Dev. (mm)	Range (mm)	U	p
Frontal Male	41	2.954	1.135	1.000 – 7.000	212.00	0.001
Female	21	2.019	0.966	0.000 – 4.300		
Occipital Male	37	3.573	1.462	0.800 – 7.800	267.00	0.236
Female	18	2.972	1.476	0.000 – 5.600		
Right euryon Male	42	1.838	1.128	0.000 – 4.500	401.00	0.968
Female	18	1.961	1.123	0.000 – 4.700		
Left euryon Male	41	1.724	1.162	0.000 – 4.900	392.00	0.710
Female	19	1.537	1.008	0.000 – 3.600		

Tables 2 and 3 show the Pearson correlation matrices for the correlation between the diploeic thicknesses at the four sample points by sex. For males there is both a significant correlation between frontal and occipital thickness (p = 0.028), as well as between the left and right euryon (p < 0.001). The latter is barely the case for females (p = 0.055).

**Table 2:** Pearson correlation matrix for diploeic thickness for males. Probabilities are Bonferroni adjusted probabilities.

	Frontal		Occipital		Right euryon		Left euryon	
	corr. Coef. p		corr. coef. P		corr. coef. p		corr. coef. p	
Frontal	1.000	0.0						
Occipital	0.473	0.028	1.000	0.0				
Right eu	0.312	0.433	0.138	1.000	1.000	0.0		
Left eu	0.324	0.370	0.177	1.000	0.712	0.000	1.000	0.0

**Table 3:** Pearson correlation matrix for diploeic thickness for females. Probabilities are Bonferroni adjusted probabilities.

	Frontal		Occipital		Right euryon		Left euryon	
	corr. Coef. p		corr. coef. P		corr. coef. p		corr. coef. p	
Frontal	1.000	0.0						
Occipital	0.352	1.000	1.000	0.0				
Right eu	0.336	1.000	0.137	1.000	1.000	0.0		
Left eu	-0.113	1.000	-0.017	1.000	0.628	0.055	1.000	0.0

The thickness of the diploe was highly correlated with total cranial thickness at all sampling points, except at the left for females (table 4).

**Table 4:** Pearson correlations for diploeic and total cranial thickness.

	Males		Females	
	r	p	r	p

Frontal dip. vs. total	0.759	0.000	0.729	0.038
Occipital dip. vs. total	0.584	0.008	0.771	0.013
Right euryon dip. vs. total	0.915	0.000	0.845	0.001
Left euryon dip. vs. total	0.871	0.000	0.451	1.000

**Figure 3:**
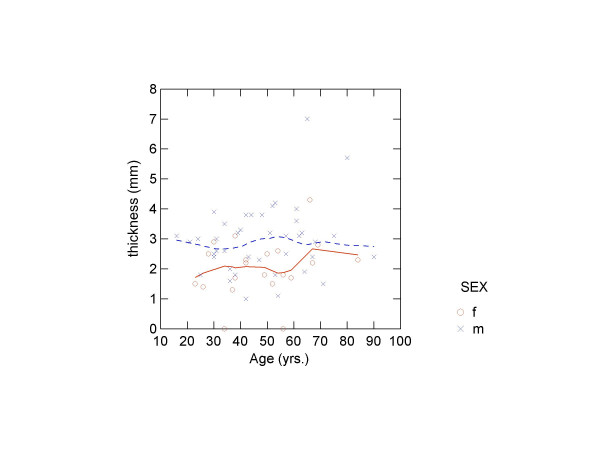
Scatter plot showing frontal diploeic thickness against age (o = females, x = males) with LOWESS smoothing (unbroken line = females; broken line = males).

**Figure 4:**
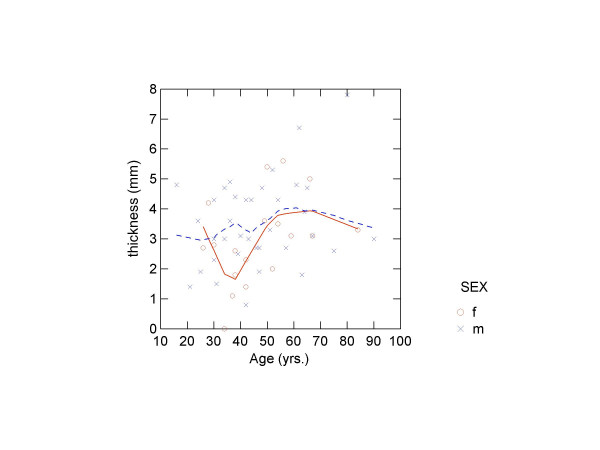
Scatter plot showing occipital diploeic thickness against age (o = females, x = males) with LOWESS smoothing (unbroken line = females; broken line = males).

**Figure 5:**
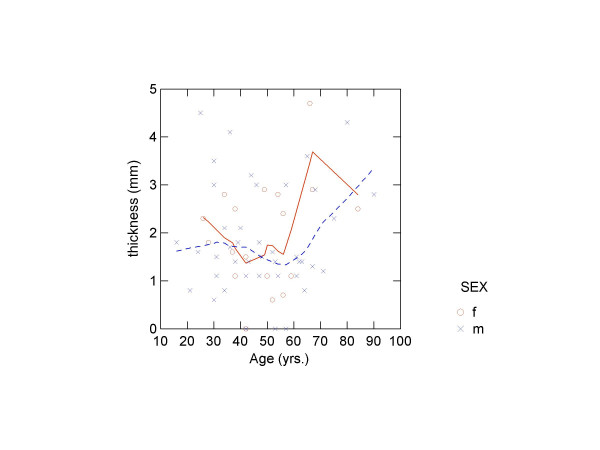
Scatter plot showing diploeic thickness at right euryon against age (o = females, x = males) with LOWESS smoothing (unbroken line = females; broken line = males).

**Figure 6:**
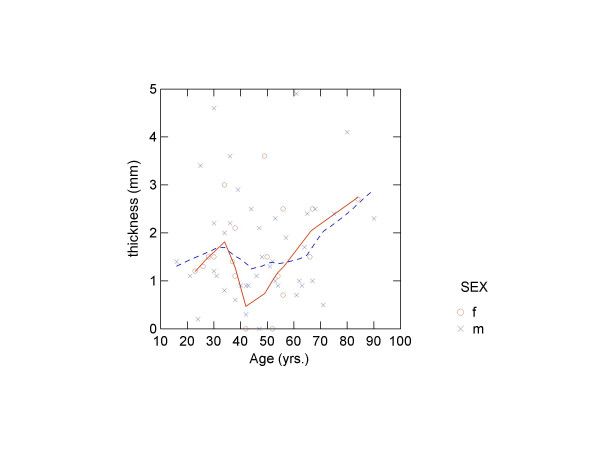
Scatter plot showing diploeic thickness at left euryon against age (o = females, x = males) with LOWESS smoothing (unbroken line = females; broken line = males).

There was no correlation between diploeic bone layer thickness and age, height or weight (table 5). Finally, LOWESS-smoothed scatter plots were produced of the four measures by age by sex (figures 3, 4, 5, 6). The plots did show a slight trend for increase in diploeic bone thickness at the left and right euryon (figures 5 and 6) for both males and females. However, the increase is first apparent above approximately 60 years of age and there is a high degree of variation when looking at the single datapoints.

**Table 5:** Pearson correlation coeficients and associated probabilities between diploeic thickness and age, height and weight. Correlation tests were performed individually for each sampling point.

	Age		Height		Weight	
	corr. coef. p		corr. coef. p		corr. coef. p	
Frontal males	0.207	1.000	-0.172	1.000	-0.062	1.000
females	0.254	1.000	0.187	1.000	0.270	1.000
Occipital males	0.293	0.470	-0.280	0.556	-0.132	1.000
females	0.398	0.610	-0.107	1.000	-0.209	1.000
Right euryon males	0.062	1.000	-0.062	1.000	-0.018	1.000
females	0.265	1.000	-0.222	1.000	-0.221	1.000
Left euryon males	0.117	1.000	-0.030	1.000	-0.082	1.000
females	0.233	1.000	-0.233	1.000	-0.175	1.000

## Discussion

We have earlier investigated total cranial vault thickness in relation to age, height and weight in a Danish forensic sample [11]. We did not then find any statistically significant correlations at the four sampling points (frontal, occipital and left and right euryon) with sex. However, that study was made by direct measurement on trephined samples, and we did not investigate the relationship between the compact bone layers and the diploe.

As in our previous study, the cranial biopsies were measured without any knowledge of the medical data of the deceased, and only the cases with cranial trauma were a priori excluded, due to the forensic pathological exigencies in these cases. When the medical data was accessed, there were no cases with diseases of the bone or bone metabolism. However, a large part of the material consisted of individuals with a known history of drug and alcohol abuse (also in several cases the direct cause of death). Chronic drug and alcohol abuse may derange bone metabolism [25] resulting in bone mass reduction [26,27] and impairment of osteoblastic actitvity [28]. Moderate levels of consumption, on the other hand, seems to correlate positively with central and peripheral bone mineral density [25]. We found no statistically significant differences when comparing the data on the diploeic thickness from individuals with drug abuse with the rest of the material.

The diploeic thickness was difficult to measure on some biopsies (table 1), as the demarcation between the diploe (cancellous or spongy bone) and the compact bone of theexternal and internal table of the cranial vault was uncertain. The thickness could not be measured for 19 biopsies (as opposed to determining that there was no cancellous bone, which was the case with 10 samples). The 19 samples with undeterminable demarcation of the diploe were not distinct in terms of age and sex from the sample as a whole.

Our main finding was a statistically significant sexual dimorphism in diploeic thickness in the frontal region, with males having a thicker diploe than females. The difference is though not directly applicable as a sex indicator, e.g., for the physical anthropologist dealing with human remains, due to the rather large overlap between males and females. The total bone thickness was not different as already determined in our previous study [11]. Males also had a thicker diploe in the occipital region and at the left euryon than female skulls, but these differences were not statistically significant. The thickness of the diploe was somewhat correlated between left and right euryon (statistically significantly so only for males), and between frontal and occipital sampling points for males. This indicates that no general statements may be made on the overall diploeic thickness of a skull.

Several authors have recorded a slight increase in cranial thickness with age and have related the frontal bone thickness increase to hyperostosis frontalis interna [4,5,10,29], while other results, also showing age-related increase in thickness [20], were later ascribed to inconsistencies in the radiologic examination [21]. It is assumed that hyperostosis frontalis interna is caused by a prolonged oestrogen production in modern (20^th ^century) females [6,30]. Ross et al. [10] found a 10% frequency of hyperostosis frontalis in females, but this is not supported by our study. We found no statistically significant correlation between age and diploeic thickness, thus reflecting previous results concerning total cranial thickness [5,9-11]. The LOWESS plots generally seem to indicate more variation with age in the frontal and occipital region for both males and females, whereas there is a slight increase in diploeic thickness with age at the left and right euryon. Calculating an index of diploeic and total thickness did not show any age-related or sexual dimorphic trends.

## Conclusion

Based on our studies we thus find that neither cranial diploeic thickness nor cranial total thickness is statistically significantly associated with the sex, weight or stature of an individuals. The diploeic bone thickness covaries with the total thickness. Powerfully built individuals may in fact have rather thin cranial vaults, whereas small, slightly built people may have thick skulls. Since the degree of cranial fracturing due to external force has been related to cranial thickness and bone structure (see e.g. Gurdjian et al. [31]), this may have implications in a forensic pathological setting, as well as in biomechanic modelling of the cranial vault [32,33].

This study, as well as many of the previous studies in this area, relies on measuring single biopsies sampled at specific locations on the cranial vault. More data, and especially more complete data might be produced, if the dimensionalities of the diploe (thickness, total volume, etc.) was calculated from serial CT or MR scans.

## Competing interests

The author(s) declare that they have no competing interests.

## Authors' contributions

NL conceived the study and its design and produced the biopsies used in the study, performed the statistical analyses and wrote the manuscript drafts. JA performed all measurements and digital X-ray image acquisition and participated in the statistical analyses. BS set up the digital X-ray acquisition as well as measurement calibration. All authors read and approved the final manuscript.
